# Understanding determinants of nutrition, physical activity and quality of life among older adults: the Wellbeing, Eating and Exercise for a Long Life (WELL) study

**DOI:** 10.1186/1477-7525-10-109

**Published:** 2012-09-12

**Authors:** Sarah A McNaughton, David Crawford, Kylie Ball, Jo Salmon

**Affiliations:** 1 Centre for Physical Activity & Nutrition Research, School of Exercise & Nutrition Sciences, Deakin University, 221 Burwood Hwy, 3125 , Burwood, VIC, Australia

## Abstract

**Background:**

Nutrition and physical activity are major determinants of health and quality of life; however, there exists little research focusing on determinants of these behaviours in older adults. This is important, since just as these behaviours vary according to subpopulation, it is likely that the determinants also vary. An understanding of the modifiable determinants of nutrition and physical activity behaviours among older adults to take into account the specific life-stage context is required in order to develop effective interventions to promote health and well-being and prevent chronic disease and improve quality of life.

**Methods:**

The aim of this work is to identify how intrapersonal, social and environmental factors influence nutrition and physical activity behaviours among older adults living in urban and rural areas. This study is a cohort study of adults aged 55-65 years across urban and rural Victoria, Australia. Participants completed questionnaires at baseline in 2010 and will complete follow-up questionnaires in 2012 and 2014. Self-report questionnaires will be used to assess outcomes such as food intake, physical activity and sedentary behaviours, anthropometry and quality of life. Explanatory variables include socioeconomic position, and measures of the three levels of influence on older adults’ nutrition and physical activity behaviours (intrapersonal, social and perceived environmental influences).

**Discussion:**

Obesity and its determinant behaviours, physical inactivity and poor diet are major public health concerns and are significant determinants of the quality of life among the ageing population. There is a critical need for a better understanding of the determinants of nutrition and physical activity in this important target group. This research will provide evidence for the development of effective policies and programs to promote and support increased physical activity and healthy eating behaviours among older adults.

## Background

Worldwide, it is well-recognised that the population is ageing and that this will have significant economic and social impacts. In 2009, 21% of the population in developed countries were aged>60 years and it is projected that by 2050, the proportion aged >60 years will have increased to 33%, double that of children under 15 years of age [1]. Since 1995, Australia’s estimated population aged 45 years and over has increased by 30%. While life expectancies are increasing, there is also an awareness of the need for improved quality of life at older ages. The disease burden attributable to chronic disease increases substantially from age 45 onwards, however an estimated 80% of health problems associated with old age can be prevented or delayed primarily by lifestyle changes implemented in the 55-65 year age group [2].

### Nutrition, physical activity and ageing

Nutrition and physical activity are major determinants of health and disease and are associated with risk of premature mortality, coronary heart disease, hypertension, colon cancer, type 2 diabetes, osteoporosis and weight gain [3]. Promoting physical activity and a healthy diet thus has the potential to substantially reduce the burden of disease and improve quality of life. Currently older adults consume too few fruits and vegetables, and have lower than recommended intakes of a range of nutrients important for prevention of chronic disease [4]. It is also estimated that approximately 45% of adults are not sufficiently active to achieve health benefits and older adults are less likely to participate in ‘sufficient’ physical activity than younger adults [5].

There are a number of specific issues relevant to nutrition and physical activity behaviours of older adults. Nutritional needs change during older age with the required intakes of many nutrients increasing alongside a decreased energy requirement [6]. Therefore, the quality of diet with food choices based on nutrient-dense foods becomes increasingly important, particularly for the avoidance of weight gain. In addition, there is an increasing use of medications with potential for interactions with dietary intake and nutritional status [6]. Of particular significance with respect to physical activity, age-associated loss of muscle mass can result in reduced muscle strength in older persons [7] and is a major cause of their increased disability prevalence [8]. Increased physical activity is a potentially important strategy among older adults for maintaining functional status and independence [9]. Later adulthood is a critical period for promotion of nutrition and physical activity, as chronic disease will typically present during this life-stage, there are immediate benefits to improving chronic disease risk profiles and there is an ability to maximize health by avoiding or delaying preventable disability [3].

As well as the biological changes that accompany ageing, it is a period of social and psychological transition. During older adult life, there are a number of transitions that can lead to substantial lifestyle changes which may directly or indirectly impact on health including retirement, relationship breakdown or partner loss and changing household structures (“empty nest”). Populations undergoing transitional life-stages are at increased risk of poor health due to potential changes to lifestyle that impact negatively on nutrition and physical activity behaviours [10,11]. A life-course approach to prevention is necessary to develop interventions that are relevant to each stage of life, with strategies that are age-appropriate [3]. Existing research in the area of health and well-being of older adults focuses on the predictors or risk factors for chronic disease and use of health services [12] and there is little research focusing on the influences of nutrition and physical activity behaviours. In addition, there are few studies assessing nutrition and physical activity behaviours longitudinally among older adults. Longitudinal research is important for enabling tracking of behaviours and their determinants during this period of potential transition and the development of causal theoretical models of health behaviour.

### Socioeconomic and geographic variations in nutrition and physical activity

Socioeconomic differentials in health including those relating to obesity are well recognised [13]. Similarly, nutrition and physical activity behaviours are known to vary according to socioeconomic position. There is little research on the mechanisms underlying socioeconomic variations in nutrition and physical activity behaviours specific to the older adult population group and how socioeconomic differentials in these behaviours are impacted by the life events typical in this life-stage, such as retirement [14,15].

In addition, rural populations suffer higher rates of socioeconomic disadvantage with lower incomes, and lower levels of educational attainment. Older adults living in rural areas have worse health compared with those in cities with lower life expectancies and higher rates of illness and disease [16]. People living in rural areas face particular challenges which impact upon health, including social isolation, limited access to transport, facilities and services [16]. The rural population is particularly susceptible to the problems associated with an ageing population since rural areas have a higher proportion of older adults compared to urban areas, driven by a combination of inward migration of older adults and outward migration of young people [16].

### Understanding nutrition and physical activity behaviours in ageing

A variety of models have been applied to the study of health behaviour, such as the theory of planned behaviour, social cognitive theory and the transtheoretical or “stages of change” model [17]. A broader framework is the social ecological model [18] which acknowledges the environment in which the behaviours occur [19] and that there is a need to consider the influence of factors in the social and physical environment, the inter-relationships between environmental and intrapersonal influences, and the ability of the individual to adapt to these influences.

Intrapersonal factors such as self-efficacy, enjoyment, barriers and intentions in relation to nutrition and physical activity and social influences such as social support and sabotage are thought to be important influences on nutrition and physical activity behaviours [20]. However, there is little research concerning these influences among older adults and just as nutrition and physical activity behaviours vary according to subpopulation, it is likely that the determinants also vary. For example, in cross-sectional studies of mid-aged and older adults, nutrition knowledge [21], self-efficacy, family support factors [22] and aspects of the environment have been shown to be associated with eating behaviours [23]. However existing studies focus on broad age ranges (>40 years) and are not specifically focused on older adults in the peri-retirement phase and therefore it is necessary for research on the influences on nutrition and physical activity behaviours to take into account the specific life-stage context [24]. Furthermore, conducting research in the Australian context is important for the development of appropriate strategies and interventions and may be particularly important when trying to understand interactions between intrapersonal, social and environmental influences as important cross-country variations in some determinants have been demonstrated [25].

#### Study aims

 · To examine nutrition and physical activity behaviours, obesity and quality of life among older adults aged 55-65 years and track changes in these behaviours and outcomes over 2 and 4 year periods.

 · To examine the intrapersonal, social and environmental influences on nutrition and physical activity behaviours and changes in these behaviours among older adults.

 · To assess variations in nutrition and physical activity behaviours and obesity across urban and rural areas among older adults.

 · To assess variations in nutrition and physical activity behaviours and obesity according to socioeconomic position and investigate the mechanisms through which socioeconomic position influences nutrition, physical activity and obesity among older adults.

## Methods

The study was designed as a prospective cohort study of older adults aged 55-65 years at baseline, with baseline data collection in 2010 and follow-up at two-year intervals at Time 2 (T2, 2012) and Time 3 (T3, 2014). Data at T2 and T3 will be collected at the same time of year as T1 to negate any potential seasonal effects. Data is collected using a self-administered postal questionnaire. Adults aged 55-65 years were the focus of this study as they are an important group with respect to chronic disease prevention and they are potentially going through a number of life-stage transitions such as retirement.

### Participants

Participants were selected from the Australian Electoral Commission (AEC) using a stratified random sampling process. In Australia, voting is compulsory for persons aged 18 years and over, and the AEC estimates that the electoral role represents 89.7% of those who were eligible to enrol and vote [26]. Suburbs in Victoria were classified as urban or rural using a classification consistent with the Australian Regional Infrastructure Development Fund Act 1999 [27] and suburbs with populations of less than 1000 or less than 200 55-65 year olds were excluded. All suburbs in urban and rural areas were then classified according to the socioeconomic Index for Areas score (SEIFA) which is assigned by the Australian Bureau of Statistics [28], and divided into tertiles (i.e low, medium and high SEIFA). Fourteen postcodes from each SEIFA tertile (i.e low, medium and high SEIFA) were randomly selected and an equal number of participants from areas from each tertile of SEIFA score selected. From each suburb, 134 participants (equal numbers of men and women) were selected, resulting in a total sampling pool of 11256. Of the surveys distributed, 380 were returned as undeliverable and 95 were returned from individuals outside of the 55-65 year age bracket. In total, 4,082 completed surveys were returned (38% response rate). Table 1 shows the sociodemographic characteristics of our final sample. 

**Table 1:** Socio-demographic characteristics of participants in the WELL study at baseline (n = 4082)

**Variables**	**Women**	**Men**
	**(n = 2138)**	**(n = 1944)**
Age (mean ± SD)	60.3 (3.2)	60.2 (3.1)
Region of residence (%)		
Urban	46.7	47.7
Rural	53.3	52.3
Education (%)		
Up to 12 years	53.6	43.9
trade/certificate	19.9	28.4
University degree	26.5	27.8
Marital status (%)		
Married/Living as married	71.4	81.7
Separated/Divorced	14.9	9.9
Widowed	6.6	2.1
Never married	4.3	6.3
Country of Birth (%)		
Australia	81.3	79.1
United Kingdom	6.7	6.2
Other	12.0	14.7
Housing tenure (%)		
Owner-occupier	89.3	88.2
Renter/boarder	10.7	11.8
Employment Status (%)		
Retired	37.3	29.0
Working full-time	20.4	47.9
Working part-time	31.9	18.7
Other	10.4	4.4
Smoking habits (%)		
Never smoker	56.5	43.5
Former smoker	37.8	42.9
Daily smoker	10.7	13.6

### Procedure

Participants selected from the electoral role were sent a letter inviting them to participate in the study and one week later were sent the survey and a reply-paid envelope for survey return. After three weeks, non-respondents received a reminder letter encouraging them to return their questionnaire. After a further three weeks, the remaining non-respondents received a second reminder letter and a replacement questionnaire and reply-paid envelope. This process of sending two reminders is standard practice [29,30].

Participants will be re-contacted at T2 and T3 and the same procedures and protocols for postal survey administration will be used. Follow-up after two and four years will allow sufficient time to detect changes in weight, nutrition and physical activity during this life-stage [31]. Recruitment and retention are promoted *via* media releases in the local survey areas, personalised survey letters, newsletters to participants with details of study results, birthday cards and access to a study website and phone number for information and change of address.

### Questionnaire

The questionnaire was designed to include measures of the outcome variables (nutrition, physical activity, sedentary behaviour, obesity, quality of life), potential determinants of these outcomes and relevant covariates. The social ecological model was used as a framework for development of the questionnaire and selection of the range of potential determinants of nutrition and physical activity behaviours [24]. Items on the questionnaire examined all three levels of influence on nutrition and physical behaviours (intrapersonal, social and neighbourhood environmental influences). Where possible, established questionnaire items from the literature with known reliability and validity were used. The full range of measures included in the questionnaire are shown in Table 2 and key variables are summarised here. 

**Table 2:** **Summary of key variables assessed*via*self-reported questionnaire in the WELL Study of adults aged >55 years**

**Biological & health-related measures**	
	Quality of life (SF36)
	Presence of physical health conditions and disability
	Menopause status (women only)
	Self-reported weight and height
Sociodemographic variables	
	Age
	Country of birth
	English language spoken at home
	Marital status
	Employment status and working hours (own and spouse)
	Retirement status
	Household composition
	Number of children and grandchildren
	Education level (own and spouse)
	Income (own and household)
	Home ownership status
	Motor vehicle access
	Role as a carer
Health behaviours	
	Physical activity (leisure, transport, domestic, occupational)
	Time spent sitting
	Frequency of food intake (111 food items)
	Eating behaviours (breakfast consumption, salt use, type of milk consumed, trimming the fat from meat, daily fruit and vegetable consumption)
	Food security
	Self-weighing frequency
	Smoking
Potential determinants of nutrition and physical activity	
Intrapersonal factors	Nutrition knowledge
	Outcome expectancies
	Self-efficacy
	Perceived behavioural control
	Perceptions of retirement
	Barriers and intentions
Social factors	Social support from family and friends
	Social norms
	Social capital, social cohesion
	Social participation
Home and Neighbourhood environmental factors	Perceptions of neighbourhood (safety, aesthetics, walking environment)
	Number of televisions
	Home availability of fruits, vegetables and high energy foods and beverages
	Perceptions of cost, availability and quality of food in neighbourhood

### Quality of life

The Medical Outcomes Study Short-Form General Health Survey (SF-36) is included as a measure of quality of life [32-34]. Scores for General Health, Physical Health, and Mental Health are computed. The Physical Health Component includes physical functioning, role-physical, bodily pain, and general health. The Mental Health Component includes vitality, social functioning, role-emotional and mental health. The questions were altered to Australian conditions in line with the Australian Longitudinal Study on Women’s Health [33,35,36].

### Anthropometry

Measures of self-reported height and weight were collected. Self-reported height and weight data are strongly correlated with measured height and weight *r* > 0.9 [37]. Self-reported weight and height information is adequate for use in large epidemiological studies examining weight or body mass index [38-40] and has been used in several large cohorts in Australia and worldwide to investigate weight change [41,42].

### Dietary intake

Diet was measured using a 111-item food frequency questionnaire assessing usual frequency of intake of food and beverages over the last 6 months previously developed for use with Australian adults in the National Nutrition Survey and other national surveys [43-45]. Additional validated short questions on food habits concerning breakfast consumption, salt use, type of milk consumed, trimming the fat from meat, daily fruit and vegetable consumption and food security were also included [45,46].

### Physical activity and sedentary behaviours

Physical activity in the past week was assessed using the long version of the self-administered International Physical Activity Questionnaire (IPAQ-L). This survey demonstrated excellent one-week test-retest reliability (pooled *r* = 0.81) and acceptable validity (pooled *r* = 0.33) when compared to accelerometer-measured physical activity in a 12-country, 14-site study [47]. The IPAQ-L assesses duration, frequency and intensity of leisure, work, commuting and household/yard activities. Data on total sitting time were also collected from the IPAQ-long with respondents asked to report time spent sitting while at work, at home, while doing study, and in leisure-time during the last 7 days [47,48] Respondents were also asked to report sitting time while doing specific activities (watching tv and during computer activities) [49].

### Sociodemographic factors

Demographic variables that were considered to be important potential moderators or confounders of the associations between behavioural predictors and outcomes were measured. These included age, country of birth, marital status, measures of socioeconomic position (education, employment, own and household income, postcode as an area level measure of socioeconomic position) [50,51], retirement status, household composition and living arrangements.

### Intrapersonal factors

In relation to nutrition and physical activity, the questionnaire included measures of self-efficacy [52], enjoyment, barriers and intentions [53,54], outcome expectancies[55], perceived behavioural control [54] and nutrition knowledge [56]. It also included measures of perceptions of ageing and retirement [57].

### Social factors

The questionnaire assessed support and sabotage for nutrition and physical activity behaviours (i.e. family and friend support and sabotage) [58], social participation [59], social capital [60] and social cohesion [61] using established measures.

Environmental influences (home and neighbourhood): Participants were asked about their perceptions of their local environment including safety, aesthetics, walking environment [62], and the cost, availability, and convenience of food and food stores. Home availability of fruits, vegetables and high energy foods and beverages and the number of televisions in the house were also assessed.

#### Analysis

Data will be initially analysed using univariate statistics to examine the distribution of key variables. Based on the initial descriptive analyses, we will employ multivariate procedures where appropriate to examine the correlates of nutrition and physical activity behaviours. We will systematically examine associations between the different domains of intrapersonal, social and environmental characteristics; and physical activity and food intake behaviours. Urban-rural, and socioeconomic comparisons in key outcomes and determinants and their associations will be examined using t-tests, ANOVA and regression models with interaction terms. We will conduct longitudinal regression analysis using baseline measures of intrapersonal, social and neighbourhood environmental factors to test predictive models of behaviour and investigate the effect of changes in nutrition and physical activity behaviours on weight status and quality of life. Multilevel modelling will be used to take into account the effect of area-level measures of socioeconomic status and environment. In addition, the mediating relationships among intrapersonal, social and environmental factors and nutrition and physical activity behaviours and obesity will be examined using structural equation modelling and mediational techniques based on regression analyses [63].

#### Ethics and study funding

Ethical approval to conduct the study was granted by the Deakin University Human Research Ethics Committee (2009-105). This project was awarded funding from the Diabetes Australia Research Trust for the baseline measures in the urban sample of participants. Funding was also received from the Australian Research Council to establish the rural sample and for the two-year follow-up (T2) and the four-year follow-up (T3) of both groups (Project Grant ID: DP1095595, FT100100581).

## Discussion

Obesity and its determinant behaviours, physical inactivity and poor diet are major public health concerns and are significant determinants of the quality of life among the ageing population. However, influences on eating and physical activity behaviours among older adults are currently not well understood. This cohort has a number of unique features that will allow the development of a thorough understanding of the determinants of nutrition and physical activity behaviour, obesity and quality of life among older adults. For example, it will focus on adults aged 55-65 years, a sub-group of older adults likely to be undergoing a number of life transitions, particularly retirement, and therefore, are at risk of weight gain. In addition, longitudinal data on physical activity and food intake in older adults will be gathered allowing changes in diet and physical activity to be tracked in order to understand the changes in nutrition and physical activity behaviours during this stage of transition.

The promotion of nutrition and physical activity is a key strategy for the prevention of a range of chronic diseases including cardiovascular disease, obesity, diabetes mellitus and cancer, as well as osteoporosis, asthma and poor mental health, and has the potential to substantially reduce the burden of disease in Australia. Improving nutrition and physical activity is likely to have significant economic benefits for Australia, with long-term gains in productivity and reductions in both direct and indirect healthcare costs [64]. While much is known about the importance of these lifestyle behaviours in health and disease, little is known about the optimal strategies for their promotion among older adults. This research will contribute evidence on key behavioural determinants which is required in order to inform the development of effective policies and programs to promote and support increased physical activity and healthy eating behaviours among older adults.

## Competing interests

The authors declare that they have no competing interests.

## Authors’ contributions

SAM took a leading role in writing the grant that was subsequently funded by the Diabetes Australia Research Trust and the Australian Research Council and drafting the study protocol for publication. All authors contributed to the study design, grant preparation, questionnaire and measures development and contributed to the drafting and editing of the manuscript. All authors read and approved the final manuscript.
